# Physics-Informed Generative Framework to Unsupervised Biomechanical Parameter Estimation for Tool–Tissue Force Prediction from Laparoscopic Depth Maps

**DOI:** 10.3390/bioengineering13080863

**Published:** 2026-07-26

**Authors:** Fabiano Bini, Alessia Finti, Guido Manni, Franco Marinozzi

**Affiliations:** 1 Department of Mechanical and Aerospace Engineering, Sapienza University of Rome, 00184 Rome, Italy; alessia.finti@uniroma1.it (A.F.); franco.marinozzi@uniroma1.it (F.M.); 2 Institute of Bioimaging and Complex Biological Systems, National Research Council, 90015 Cefalù, Italy; 3 Unit of Artificial Intelligence and Computer Systems, Department of Engineering, Università Campus Bio-Medico di Roma, 00128 Rome, Italy; Guido.Manni@unicampus.it

**Keywords:** physics-informed neural networks, surgical simulation, force estimation, soft tissue mechanics, minimally invasive surgery, biomechanical parameter identification, depth estimation, laparoscopic surgery

## Abstract

Physically consistent estimation of soft-tissue mechanical properties is critical for surgical robotics, intraoperative safety monitoring, and simulator initialization, yet existing methods typically require force-sensing hardware or manual parameter tuning. This paper presents a physics-informed generative framework that estimates tissue stiffness ($k_{s}$), damping coefficient ($k_{d}$), and tool–tissue contact force magnitude ($F_{mag}$) from monocular laparoscopic video in a label-free manner with respect to mechanical parameters and interaction forces. The pipeline integrates three components: DepthPro, a multi-scale Vision Transformer (ViT) for zero-shot metric depth estimation; a 3D geometric contact detection pipeline; and a dual-mode conditional generative network trained via a five-term physics–adversarial loss. A differentiable Mass–Spring–Damper (MSD) simulator is embedded directly in the training loop. This enables gradient-based parameter learning without force-sensor, displacement, or boundary-condition supervision. Parameter identifiability is supported through dual observational grounding: MSD physics consistency against observed contact displacement, and next-frame depth map reconstruction. Validated on CholecSeg8k cholecystectomy sequences, Physics-Informed Neural Network (PINN)-estimated parameters significantly outperform static literature baselines (Wilcoxon *p* = 2.49 × 10^−8^, Cohen’s d = 0.374), with physically plausible viscoelastic settling dynamics recovered within 0.9 s of tool release. Since no force sensors were present at acquisition time, evaluation follows an indirect simulation-consistency protocol. Mechanical parameters are estimated at 1.6 ms/frame, a negligible addition to the monocular depth front end that sets the pipeline rate. Estimated parameters directly enable stiffness-aware haptic rendering, intraoperative safety monitoring, and scene-adapted surgical simulation initialization.

## 1. Introduction

Soft biological tissues exhibit complex mechanical behavior that varies substantially across anatomical structures, pathological states, and individual patients [1]. Characterizing this behavior through quantifying stiffness, damping, and the forces exchanged at tool–tissue interfaces is a challenge in biomedical engineering with direct implications for surgical safety, robotic system design, and the development of physically realistic simulation environments [2,3]. In open surgery, the surgeon’s hand provides continuous tactile feedback that implicitly encodes tissue compliance and contact force, enabling real-time adaptation of manipulation strategy. This biomechanical sensing channel is entirely absent in minimally invasive surgery (MIS), where instruments and robotic interfaces place a physical and perceptual barrier between the operator and the tissue [4].

The clinical consequences of this absence are well documented: without haptic information, surgeons cannot reliably distinguish healthy from pathological tissue by palpation, cannot directly modulate grip force to account for tissue fragility and cannot detect imminent perforation or tearing from mechanical overload [2,5]. A systematic review of tool–tissue forces in surgery identified excessive force application as a primary mechanism of iatrogenic injury in laparoscopic and robot-assisted procedures and noted that safety thresholds for interaction forces remain poorly characterized in the intraoperative setting precisely because measurement is technically demanding [2]. Addressing this gap requires methods capable of estimating tissue mechanical parameters and contact forces from data that are routinely available in the operating room, namely, endoscopic video.

The present work addresses this gap directly. We propose a physics-informed generative framework that estimates tissue stiffness ($k_{s})\;$ damping ($k_{d}$) and tool–tissue contact force magnitude ($F_{mag}$) from laparoscopic depth maps without requiring any force-sensor, displacement, or parameter labels at training or inference time, a capability that neither existing PINN-based tissue mechanics approaches nor vision-based force estimation methods achieve on unlabeled intraoperative data. The framework combines monocular metric depth estimation via a foundation vision model, a 3D geometric pipeline for contact geometry reconstruction, and a dual-mode conditional generative network trained through a physics–adversarial loss that enforces consistency with the Mass–Spring–Damper (MSD) governing equation. All supervision derives from the video itself: the network must simultaneously explain the observed tissue deformation through MSD simulation and reproduce the next video frame through depth map prediction. This dual observational grounding is the mechanism by which physically meaningful parameters emerge from unlabeled surgical video.

Our key contributions are:

- Scene-adapted biomechanical parameter estimation from intraoperative video: We present a framework that estimates tissue-specific viscoelastic parameters ($k_{s}$,$k_{d}$) and contact force magnitude ($F_{mag}$) directly from standard laparoscopic image sequences, without any ex vivo calibration, force sensors, or manual annotation.
- Label-free biomechanical parameter learning via dual observational grounding. Physical parameters are constrained by two independent implicit supervision signals, MSD physics consistency against observed contact displacement and next-frame depth map reconstruction, which together prevent degenerate parameter solutions without labeled ground truth.
- Complete 3D contact geometry pipeline from monocular depth: A full geometric processing chain, including metric depth estimation via DepthPro [6], point-cloud back projection, KD-tree contact detection, triangulated mesh generation, and area-weighted contact normal computation, reconstructs the contact geometry required for differentiable biomechanical simulation from a single RGB camera.
- Differentiable physics simulation enabling gradient-based parameter learning: MSD dynamics are embedded as a differentiable simulator within the training loop, enabling gradient flow through 200 integration steps back to the parameter estimation network, without requiring any ground-truth force or stiffness labels.
- Indirect evaluation on real intraoperative cholecystectomy video: On CholecSeg8k [7], acquired without force-sensor instrumentation, the estimated parameters significantly reduce simulation error relative to fixed literature values (W = 3658, *p* < 10^−6^; Cohen’s d = 0.390) and produce a physically plausible viscoelastic settling, with equilibrium restored within 0.9 s of tool release. Absent force sensors: evaluation follows an indirect simulation-consistency protocol; parameter estimation adds 1.6 ms/frame to a pipeline whose rate is set by the depth front end.

The novelty of the proposed framework lies not in any individual component, as monocular depth estimation, differentiable physics simulation, and generative learning have each been investigated independently, but in their integration within a unified architecture trainable on unannotated in vivo laparoscopic video. No existing PINN-based approach for soft-tissue mechanics operates without known forcing terms, prescribed boundary conditions, or labeled mechanical properties at training time [8,9,10,11,12], and no existing vision-based force estimation method can be trained or validated on unlabeled in vivo surgical data, as all reported methods require synchronized force-sensor measurements [13,14,15,16]. The dual observational grounding mechanism introduced here, constraining predicted parameters simultaneously via physics-consistency loss against observed contact displacement and via depth-reconstruction loss against the observed next frame, is the structural contribution that enables unsupervised parameter identifiability without any form of mechanical ground truth. To the authors’ knowledge, this is the first pipeline enabling label-free viscoelastic parameter and force estimation directly from standard clinical laparoscopic video.

This paper is organized as follows: Section 2 reviews related work on tissue mechanical characterization, computational tissue modeling, and physics-informed and vision-based approaches. Section 3 describes the complete Materials and Methods, covering the dataset, the 3D geometric pipeline, the MSD physics model, and the PINN architecture and training procedure. Section 4 presents the experimental results, including quantitative validation, dynamic tissue response analysis, and computational performance. Section 5 addresses limitations and future directions. Section 6 concludes this paper.

## 2. Related Work

Tissue mechanical characterization in biomedical engineering has traditionally relied on ex vivo rheometry, indentation testing, or imaging-based elastography [7,17,18]. These methods yield population-level parameter estimates that may not reflect the state of tissue being operated on in a specific patient. Intraoperative variability in tissue stiffness and damping is substantial: inflammation, tumor infiltration, fibrosis, and surgical manipulation itself alter the mechanical response of soft tissue in ways that fixed literature values cannot capture [19]. What is needed is a method that estimates patient-specific biomechanical parameters from the surgical scene itself, in real time, without requiring instrumentation beyond the standard laparoscopic imaging chain.

Computational approaches to this problem are broadly divided into physics-based and learning-based families. Finite element method (FEM) models with hyperelastic constitutive laws provide high-fidelity tissue deformation predictions but require a priori knowledge of material parameters and impose computational costs incompatible with real-time surgical use [17,20]. Lightweight alternatives such as MSD systems can run in real time and have been used extensively in surgical simulation [19,21], but their stiffness and damping parameters are typically set to fixed literature values that do not adapt to the patient. Both families share a deeper limitation: they require boundary conditions, forces or displacements imposed by the surgical instruments, that cannot be measured from standard intraoperative video. This bottleneck has been explicitly identified in recent systematic reviews as a principal obstacle to clinical deployment of biomechanical models for surgical navigation [22].

PINNs offer a data-driven path around this bottleneck by embedding differential equations directly into the loss function, enabling joint learning of system dynamics and the underlying physical parameters that govern them [9,10]. In biomechanics, PINNs have been applied to displacement-field reconstruction and material property estimation in soft tissue [8], noninvasive inference of cardiac valve elastic properties from echocardiogram sequences [18], musculoskeletal parameter identification [23], 3D soft-tissue dynamics prediction from 2D images [24], and real-time deformation modeling via PINN–neural ODE frameworks coupled with mass–spring systems [11]. In surgical contexts, PINNs have been used to embed finite-element elasticity constraints into augmented reality tracking [12], and hybrid variants including physics-informed Generative Adversarial Networks (GANs) [25] and mixed-material simulation networks [26] further extend this paradigm towards broader generative AI frameworks and graph-based neural abstractions [27,28].

However, a critical limitation shared by existing PINN-based and vision-based approaches is their dependence on supervisory signals that are unavailable in routine clinical practice. Most methods assume known forcing terms, prescribed boundary conditions, or labeled mechanical properties at training time [9,10,25]. Vision-based force estimation methods [13,14,15,16] recover interaction forces from visual input but require synchronized ground-truth measurements from load cells, force-sensing trocars, or fiber Bragg grating sensors, and are therefore validated exclusively on phantoms, ex vivo preparations, or instrumented robotic platforms. The structural consequence is that none of these approaches can be trained or validated on unannotated intraoperative video, the only data modality that is universally available across surgical settings.

## 3. Materials and Methods

**Figure 1:**
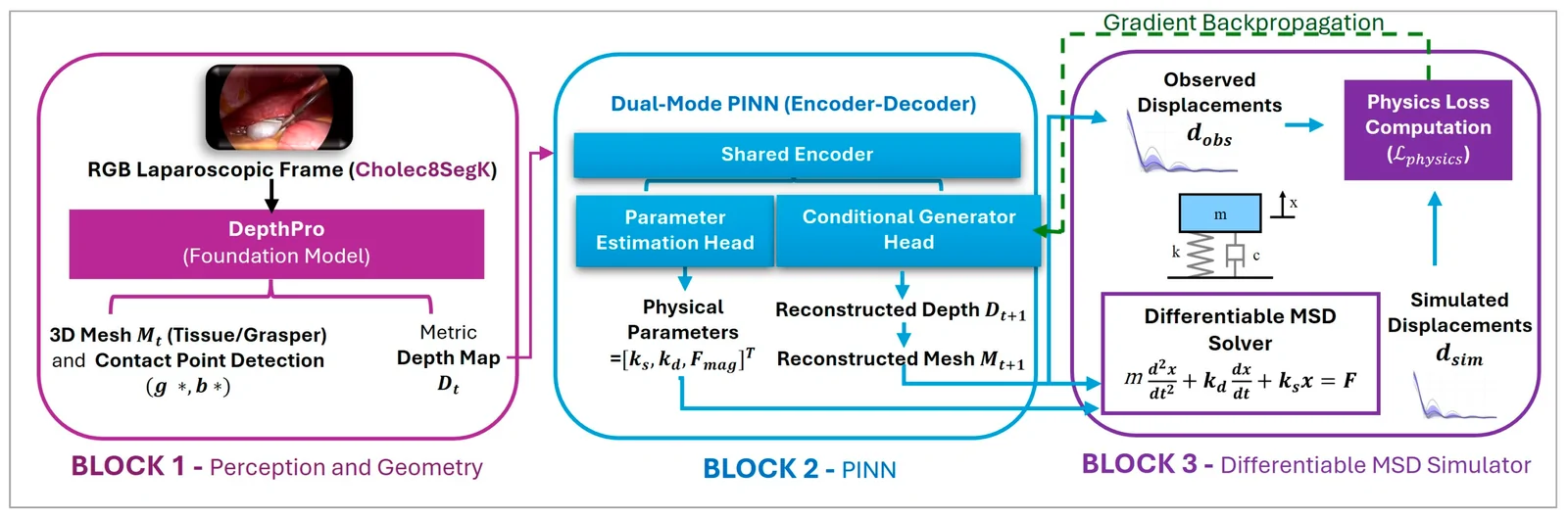
Overview of the proposed methodological framework. The architecture is divided into three main stages: Block 1 extracts metric depth maps and 3D meshes from laparoscopic RGB frames using the *DepthPro* foundation model; Block 2 employs a Dual-Mode PINN (Encoder-Decoder) to estimate physical parameters (θ) and reconstruct the deformed geometry; Block 3 integrates a differentiable MSD solver to compute the physics loss $L_{physics}$, which is then backpropagated to optimize the network, ensuring adherence to physical laws.

The proposed framework operates through a sequential three-stage pipeline (Figure 1). First, during laparoscopic surgery, monocular metric depth maps are generated in real time from endoscopic video frames using a foundation depth estimation model (Section 3.2.1). Second, a geometric processing pipeline reconstructs the 3D contact geometry, including tool and tissue point clouds, contact detection, triangulated mesh, and contact normal estimation (Section 3.2.2). Third, consecutive depth maps encoding tissue displacement are fed into a physics-informed neural network that jointly estimates the biomechanical parameters, stiffness ($k_{s}$) and damping ($k_{d})\;$and the magnitude of the tool–tissue interaction force $\left(F_{mag}\right)$ (Section 3.4). These predicted quantities are passed to an embedded MSD model that simulates the expected tissue deformation (Section 3.3).

### 3.1. Dataset

Experiments were conducted on CholecSeg8k [7], a publicly available pixel-level semantic segmentation benchmark for laparoscopic cholecystectomy derived from the Cholec80 video collection [29]. It encompasses 8080 frames drawn from 17 surgical video clips, each labeled across thirteen anatomical and instrument classes at native resolution of 854 × 480 pixels. The class taxonomy was defined around the anatomical targets of cholecystectomy, with grasper (class 5) and gallbladder (class 10) being the two classes central to this work.

A curated subset of 1190 frames spanning 10 videos was retained by restricting selection to clips that exhibited active tool–tissue contact and tissue deformation attributable to mechanical loading rather than to rigid-body camera motion or global scene displacement. Partitioning followed a video-level stratification strategy to eliminate temporal leakage between splits: 837 frames from 7 videos constituted the training set (70%), 177 frames from 2 videos the validation set (20%), and 176 frames from the remaining video the held-out test set (10%).

Before network training, each RGB frame was converted to a dense depth map through DepthPro and linearly normalized. The resulting maps D(u,v)∈ [0, 1] encode per-pixel depth relative to the scene, with u and v indexing pixel columns and rows, respectively; they capture tissue geometry and surface displacement information unavailable in raw color data. All subsequent geometric and mechanical computations are carried out in this normalized space. Class-conditioned binary masks derived from the semantic annotations were used to separate tool and tissue depth channels: pixels assigned to the grasper class (5) yielded $D_{grasper}\left(u,v\right)$, and those assigned to the gallbladder class (10) yielded $D_{glbd}\left(u,v\right)$, providing the two spatially registered depth representations required by the geometric contact pipeline described in Section 3.2.2. These masks act solely as a spatial partition of the depth map: the estimation network receives the depth channels and the reconstructed contact geometry alone, never the class labels, and only the binary tool-versus-tissue distinction is used, the thirteen-class taxonomy being simply the annotation format distributed with the dataset. Reference annotations were adopted deliberately, so that the parameter estimates reported in Section 4 are not confounded by segmentation error; the substitution of lightweight automatic alternatives is addressed as future work in Section 5. Depth maps were stored as single-channel 8-bit PNG images at the native resolution of 854 × 480 pixels and linearly normalized to [0, 1] by division by 255. Each of the two structures was isolated from the depth map by color matching against its annotation color in the semantic mask, using an L1 color distance with a tolerance of 5 intensity levels; pixels whose color did not match the target were set to zero, yielding two separate masked depth maps per frame. Training samples consist of temporally consecutive frame pairs drawn from within a single video, with pairs never formed across video boundaries, giving 830 pairs for training, 177 for validation and 176 for testing.

### 3.2. Depth Estimation and 3D Geometric Pipeline

This section describes the two-stage geometric processing pipeline that converts raw laparoscopic RGB frames into the 3D contact representations required by the MSD simulator. In the first stage (Section 3.2.1), DepthPro produces a dense metric depth map from each monocular frame, providing absolute per-pixel depth without requiring camera calibration. In the second stage (Section 3.2.2), the depth map is back-projected into 3D space, and a multi-step geometric pipeline detects the tool–tissue contact point, reconstructs a triangulated mesh of the tissue surface, and computes the contact normal vector. The outputs of this pipeline, contact vertex index ${idx}^{*}$, contact normal$\;n^{*}$, contact position $g^{*}$, and observed displacement dobs, are the geometric inputs to the PINN training loop described in Section 3.4.

#### 3.2.1. Monocular Depth Estimation

The Monocular Depth Estimation Module serves as the foundational component for converting two-dimensional laparoscopic RGB frames into three-dimensional geometric representations. This module extracts dense pixel-wise depth information that directly encodes tissue displacement and contact geometry, essential for subsequent contact detection, mesh generation, and physics-informed parameter estimation.

We employ DepthPro [6], a foundation model specifically designed for zero-shot metric monocular depth estimation, building upon the principles of robust cross-domain depth transfer [30]. DepthPro was selected for several critical advantages aligned with our application requirements, and its utility has been demonstrated in other surgical imaging contexts [31]. First, it produces metric depth maps with absolute scale without requiring camera intrinsics, a crucial feature given the variability and frequent unavailability of accurate calibration data in laparoscopic settings. The metric scale is not, however, propagated to the mechanical estimates, since the maps are normalized before back-projection, and the consequences of this convention are stated in Section 3.3. Second, it generates high-resolution predictions with exceptionally sharp boundary delineation, essential for accurately capturing fine tissue structures and contact regions. Third, it operates with low latency, enabling near-real-time processing of surgical video sequences.

The DepthPro architecture employs a multi-scale Vision Transformer (ViT) design that processes the input image at multiple resolutions simultaneously. The framework consists of two main encoder components: a patch encoder and an image encoder, both utilizing plain ViT backbones with shared weights across scales. The input image is first downsampled to 1536 × 1536 resolution and split into overlapping patches of 384 × 384 pixels at different scales, yielding 35 patches in total. These patches are processed in parallel by the patch encoder, which extracts hierarchical features at multiple scales. Simultaneously, the image encoder processes the downsampled full image (384 × 384) to capture the global scene context. The multi-scale features are then merged through a Voronoi-based partitioning strategy and fused using a DPT-style decoder, which progressively upsamples and integrates features to produce the final dense depth prediction.

A key characteristic of DepthPro is its prediction of canonical inverse depth rather than direct depth values. The canonical inverse depth C is related to metric depth $D_{m}$ through the horizontal field of view: $D_{m}=(\frac{f_{px}}{w})C$, where $f_{px}$ represents the focal length in pixels and w denotes image width. This representation prioritizes accuracy in regions close to the camera, precisely where tool–tissue interactions occur in laparoscopic surgery.

#### 3.2.2. Contact Point Extraction and 3D Geometric Pipeline

Contact point extraction was performed automatically through a multi-stage geometric pipeline. Each depth map was projected into 3D space using pinhole camera geometry with intrinsic parameters from DepthPro. For each valid pixel (u,v) where D(u,v) > 0, the corresponding 3D point p=[x,y,z]ᵀ was computed as follows:

(1) $$x=(u-\;c_{x})\cdot \frac{z}{f_{x}}$$

(2) $$y=(v-c_{y})\cdot \frac{z}{f_{y}}\;$$

(3) z=D(u,v)

where x, y,z represent the 3D coordinates in the camera reference frame. This produces two point clouds: $G={{\left\{g_{i}\right\}}_{i=1}}^{Ng}\subset \;R^{3}$ representing the grasper surface, and $B={{\left\{b_{j}\right\}}_{j=1}}^{Nb}\subset \;R^{3}$ representing the gallbladder surface. Contact detection was formulated as a nearest-neighbor search problem to find the pair of closest points ($g^{*}$, $b^{*}$) between the two surfaces:

(4) $$\left(g^{*},\;b^{*}\right)=argmin{\left\|g_{i}-\;b_{j}\right\|}_{2}\;over\;all\;\left(g^{*},\;b^{*}\right)\;\in G\;\times \;B$$

To achieve computational efficiency, a KD-tree data structure was constructed from the gallbladder point cloud B, reducing the search complexity O($N_{m}\;\cdot$ $N_{\beta }$) to $\;o(N_{m}\;log\;log\;N_{\beta }$). For cases where |G| > 1000, a two-pass coarse-to-fine strategy was employed: a coarse pass using 1000 evenly sampled grasper points to identify the approximate contact region, followed by a fine pass searching all grasper points in the vicinity to determine the exact minimum-distance pair.

The contact distance ${d^{*}=\|g^{*}-\;b^{*}\|}_{2}\;$ quantifies the spatial separation between tool and tissue at the interaction site.

To enable physics-informed simulation, the gallbladder point cloud was converted into a triangulated mesh representation M = (V, F), where $V={{\left\{v_{k}\right\}}_{k=1}}^{K}\subset \;R^{3}\;$ denotes the set of K vertices and $F={{\left\{f_{m}\right\}}_{m=1}}^{M}\;$ denotes the set of M triangular faces. Each face $f_{m}=\left(a_{m},\;b_{m},\;c_{m}\right)\;$ is defined by three vertex indices. The mesh was constructed using grid-based triangulation, connecting adjacent pixels in the depth map with consistent depth values. Triangles with edge lengths exceeding 0.05 units were filtered out to prevent artifacts from depth discontinuities. A decimation step retaining 30% of vertices is available to reduce memory requirements while preserving geometric fidelity; it was not applied in the experiments reported here, as the meshes were used at full resolution.

The continuous tissue contact point $b^{*}$ ∈ $R^{3}$ was mapped to the nearest discrete vertex in the mesh:

(5) $${idx}^{*}=argmin{\left\|v_{k}-\;b^{*}\right\|}_{2}\;over\;k=1,\ldots ,\;K$$

The surface normal at the contact vertex was computed through area-weighted averaging of adjacent face normals. For each face $f_{m}\;$ incident to vertex ${idx}^{*}$, the face normal nm was calculated as follows:

(6) $$n_{m\;}=\;\frac{((v_{\beta m}-v_{am})\;\times (v_{m}^{c}-v_{am}))\;}{{\left\|(v_{\beta m}-v_{am})\;\times (v_{m}^{c}-v_{am})\right\|}_{2}\;}$$

The area $A_{m\;}$ of each face was computed as follows:

(7) $$A_{m\;}=\;\frac{{\left\|(v_{\beta m}-v_{am})\;\times (v_{m}^{c}-v_{am})\right\|}_{2}\;}{2\;}$$

The contact normal ${\;n}^{*}$ was then obtained as the area-weighted average of all adjacent face normals, normalized to unit length:

(8) $${\;n}^{*}=\;\frac{\sum A_{m\;}\cdot n_{m\;}}{{\left\|\sum A_{m\;}\cdot n_{m\;}\right\|}_{2}}\;\;\;(sum\;over\;all\;f_{m}\in T_{i}\;{d_{x}}^{*})$$

This ensures ‖${\;n}^{*}$‖_2_ = 1, yielding a unit normal vector that defines the direction of force application in the MSD dynamics formulation. To improve computational efficiency during training, images were resized from 480 × 854 to 120 × 214 pixels using bilinear interpolation, reducing memory footprint while maintaining sufficient spatial resolution for capturing tissue deformation patterns.

### 3.3. Physics Model

Tissue deformation was modeled using a Mass–Spring–Damper (MSD) formulation [32,33]. A simplified version was adopted to focus on the identification of local interaction parameters. In this model, each mesh vertex is treated as a point mass m, connected to neighboring nodes by structural springs. The internal dynamics are governed by two primary parameters, the spring stiffness $k_{s}\;$ and the damping coefficient $k_{d}$. The mass parameter m is assigned a uniform value of 0.001 kg per mesh vertex, following the infinitesimal-area approximation adopted in surface triangular mesh MSD formulations [32,33], and is held fixed throughout training, so that only $k_{s}$, $k_{d}$ and $F_{mag}$ are estimated. Since m scales all inertial terms in Equation (9) uniformly, it does not alter the relative contributions of stiffness and damping to the system dynamics, and is therefore excluded from the optimization vector θ = ${\left[k_{s},\;k_{d},\;F_{mag}\right]}^{T}.$ The simulator is fully specified by the point mass m, the integration timestep Δt, the simulation horizon T, the integrator, and the boundary condition; Table 1 reports their values. The framework is thus insensitive to the specific value of m provided it remains small relative to the contact force magnitudes.

The dynamic equilibrium of the system is defined by the sum of internal elastic and viscous forces and the external force F applied at the contact vertex:

(9) $$m\ddot{x}+\;k_{d}\dot{x}+k_{s}x=\;F_{ext}\;$$

The external force is calculated based on the estimated force magnitude ${\;F}_{mag}$ and the local surface normal ${\;n}^{*}$ (Equation (8)). The load is applied at the contact vertex over the first quarter of the simulated interval and released thereafter, so that each simulation captures both the loading phase and the subsequent free relaxation. The baseline values $\;k_{s}$ = 5.34 and $\;k_{d}$ = 0.127, drawn from the mechanical properties of human tissue characterized in [9], serve as the fixed-parameter reference for comparative evaluation and as the initialization of the optimization vector θ = ${\left[k_{s},\;k_{d},\;F_{mag}\right]}^{T}.$ The system is integrated with a semi-implicit Euler scheme at dt = 0.005 s over a horizon of T = 1.0 s, corresponding to the 200 steps traversed by backpropagation through time during training. The estimated force is parameterized as a compressive load directed along the inward surface normal, ${\;F}_{mag}$ ∈ [−10, 0], and is reported throughout as a magnitude. This MSD formulation acts as the differentiable physical constraint within the PINN framework, guiding the network to ensure that the predicted parameters result in physically consistent deformations. Because the simulator operates on the normalized geometry produced by the geometric pipeline, its constants are dimensionless and the estimated stiffness, damping and force are determined up to a single global factor fixed by the depth normalization; the units in which they are reported follow the convention of the literature values used to initialize the model rather than an independent calibration of the imaging chain. The estimates are likewise conditional on the recovered imaging geometry: the focal length enters the lateral coordinates x and y but not the depth z, so that an error in it rescales the reconstructed surface laterally while leaving depth unchanged, distorting the edge rest lengths anisotropically and propagating into the estimated stiffness. Neither dependence affects the quantities on which the framework is evaluated, namely the reduction in displacement prediction error relative to fixed parameters and the relative variation in the estimates across frames, both of which are invariant to a global rescaling; recovering absolute mechanical units would require calibrating the depth normalization against a known metric reference in the surgical field, which we identify as future work.

### 3.4. Physics-Informed Neural Network

The PINN module represents the core learning component of the framework, enabling unsupervised estimation of tissue biomechanical parameters directly from depth map sequences. The key innovation lies in embedding a differentiable MSD simulator into the training loop, which provides physics-based supervision without requiring ground-truth labels for mechanical properties. Differentiability is here a property of the implementation rather than of the governing equations alone: the integration loop of Equation (9) is composed exclusively of operations with defined derivatives and its computational graph is retained, so that the Jacobian of the simulated deformation with respect to the predicted parameters is recoverable by automatic differentiation, whereas a conventional solver of the same system would return identical trajectories without exposing this derivative. During training, the network predicts three physical parameters—spring stiffness ($k_{s}$), damping coefficient ($k_{d}$), and interaction force magnitude (${\;F}_{mag}$)—from consecutive depth maps. These parameters drive the MSD simulator to compute expected tissue deformation, and the discrepancy between simulated and observed deformation provides the supervision signal.

The architecture builds upon the dual-mode conditional generator concept introduced in CycleVO [34], originally developed for pose estimation in surgical SLAM, adapted here for biomechanical parameter estimation by reformulating the task as depth map prediction conditioned on physical parameters, where successful deformation prediction implies correct parameter estimation.

#### 3.4.1. Network Architecture

The network comprises two primary components: a dual-mode conditional generator and a patch-based discriminator. The generator performs two interconnected tasks through a shared encoder and specialized decoder heads: parameter estimation and conditional depth map generation.

The network processes single-channel depth maps $D_{t}$ ∈ R(1×H×W). The primary outputs are the physical parameters θ = ${\left[k_{s},\;k_{d},\;F_{mag}\right]}^{T},$ where $k_{s},k_{d}$ ∈ [0, 1] and $F_{mag}$ ∈ [−10, 0], and the predicted next-frame depth map $D_{t+1}$ ∈ $R^{\left(1\times H\times W\right)}.$

The encoder extracts features through an initial convolution block and two downsampling stages, producing encoded features $x_{enc}$ ∈ $R^{\left(256\times 30\times 53\right)}\;$with instance normalization and ReLU activations applied throughout. The parameter estimation head predicts ${\left[k_{s},\;k_{d}\right]}^{T}$ using sigmoid activation to constrain values to [0, 1], and $F_{mag}$ using tanh activation scaled to [−10, 0], reflecting the compressive nature of tool–tissue interactions. The conditional generation head generates ${\hat{D}}_{t+1}$ by injecting the predicted parameters θ into the encoded features, then processing them through 9 residual blocks, two upsampling stages, and a final output layer. This conditioning mechanism implements the principle that physically correct parameters enable successful reconstruction of the observed next frame.

The discriminator employs a PatchGAN architecture with four convolutional blocks (4 × 4 kernels, stride 2) and progressively increasing channel dimensions (64 → 128 → 256 → 512), producing patch-based real/fake classifications for 70 × 70 receptive fields. This patch-based approach focuses on local texture and structure discrimination, more effective than global image assessment for evaluating the realism of tissue deformation patterns. Instance normalization is employed throughout to ensure consistent behavior across the small batch size (n = 2) necessitated by memory constraints. The network contains approximately 15.2 M trainable parameters.

#### 3.4.2. Physics-Informed Loss Formulation

The training objective integrates multiple complementary objectives that balance adversarial realism, content preservation, and physical plausibility. Unlike supervised learning approaches that require ground-truth parameter labels, the framework learns in an unsupervised manner by leveraging two forms of implicit supervision: the physics-informed constraint from the MSD simulator and the reconstruction of observed depth maps. The complete optimization problem is formulated as follows:

(10) $$L_{total}=\;\lambda_{GAN}L_{GAN}+\;\lambda_{cyc}L_{cycle}+\;\lambda_{id}L_{identity}+\;\lambda_{phys}L_{physics}+\;\lambda_{reg}L_{param\_reg}$$

where the total loss comprises five terms: The adversarial loss, $L_{GAN}$, drives the generator to produce depth maps indistinguishable from real observations. The cycle consistency loss, $L_{cycle}$, enforces that forward and backward temporal transformations recover the original frame, preserving anatomical content. The identity loss, $L_{identity}$, anchors the transformation space, requiring the generator to return the input unchanged when conditioned on parameters that imply no deformation. The physics consistency loss, $L_{physics}$, constitutes the core of the framework, coupling the predicted parameters to the observed tissue displacement through the differentiable MSD simulator. Finally, the parameter regularization loss, $L_{param\_reg}$, constrains the predicted parameters to physically admissible ranges. The rest of this section details the computation of each of these five loss functions.

Adversarial loss adopts the least-squares GAN (LSGAN) formulation for improved training stability. The generator aims to produce depth maps indistinguishable from real observations, while the discriminator learns to classify real depth ($D(D_{t+1})\;\approx \;1)\;$ from generated ones $D\left(G\left(\ldots \right)\right)\approx \;0.$ The squared loss formulation provides smoother gradients compared to binary cross-entropy, contributing to more stable training dynamics. Formally, the adversarial loss is defined as follows:

(11) $$L_{GAN}(G,P,D)=\;E[{\left(D(D_{t+1})-1\right)}^{2}]+\;E[{D(G(D_{t},\;\theta_{AB}))}^{2}]$$

where the first expectation is taken over the next frames observed and the second over consecutive frame pairs.

Inspired by CycleGAN [35], the cycle consistency loss enforces that sequential forward and backward transformations recover the original input. In the temporal framework, predicting forward from frame t to t + 1 and then backward from t + 1 to t should reconstruct frame t:

(12) $$L_{cycle}\left(G,P\right)=\;\frac{1}{2}E_{\left(D_{t},\;D_{t+1}\right)}\left[{\left\|G\left(G\left(D_{t},\theta_{AB}\;\right),\;\theta_{BA}\right)-\;D_{t}\right\|}_{1}+{\left\|G(G\left(D_{t+1},\theta_{BA}\;\right)),\;\theta_{AB})\;-\;D_{t+1}\right\|}_{1}\right]$$

where $\theta_{AB}$ = $P_{AB}\left(E_{AB}\left(D_{t}\right)\right)\;$ and $\theta_{BA}$ = $P_{BA}\left(E_{BA}\left(D_{t+1}\right)\right)\;$ represent the forward and backward transformation parameters, respectively. The L1 norm is preferred over L2 because it encourages sharper reconstruction with better preservation of tissue boundaries and anatomical structures, critical for medical imaging applications. The identity loss prevents arbitrary transformations: when the generator is provided with parameters that should produce no deformation, it should return the input unchanged, acting as an anchor in transformation space. It is defined as follows:

(13) $$L_{identity}(G,P)=\;E[{\left\|G(D_{t},\;\theta_{null})-D_{t}\right\|}_{1}]$$

where $\theta_{null}$ denotes the parameter vector corresponding to zero applied force, so that the generator is required to act as the identity map when no deformation is implied.

The physics consistency loss constitutes the core innovation of the framework, directly coupling the predicted parameters with biomechanical simulation:

(14) $$L_{physics}\;(G,\;P)\;=\;E_{\left(D_{t},\;D_{t+1}\right)}\;[{\left\|\;\Phi_{MSD}\left(\theta \left(D_{t}\right);\;M_{t},{idx\;}^{*},{n\;}^{*},{g\;}^{*},T\right)-\;\;d_{obs}\left(D_{t},D_{t+1}\right)\right\|}_{2}^{2}]$$

where $\Phi_{MSD}$ represents the differentiable MSD simulator (Section 3.3), $\theta \left(D_{t}\right)\;$= ${\left[k_{s},\;k_{d},\;F_{mag}\right]}^{T}$ denotes the predicted parameters,$\;M_{t}=\;\left(V_{t,\;},\;F_{t}\right)$ is the triangulated mesh extracted from depth map $D_{t}$ (Section 3.2.2), (${idx\;}^{*},{n\;}^{*},{g\;}^{*})$ are the contact vertex index, contact normal, and contact position computed through the geometric pipeline, T is the simulation time, and $\;d_{obs}\left(D_{t};D_{t+1}\right)\;$ is the observed displacement of the contact point between consecutive frames.

The differentiability of $\Phi_{MSD}$ enables the gradient flow:

(15) $$\frac{\partial L_{physics}}{\partial \theta }=\;\frac{\partial L_{physics}}{\partial d_{sim}}\cdot \frac{\partial d_{sim}}{\partial p(T)}\cdot \;\frac{\partial p(T)}{\partial \theta }\;$$

where $p\left(T\right)$ denotes the position of the contact vertex at the final simulation time. The gradients $\frac{\partial p(T)}{\partial \theta }\;$ are computed via backpropagation through time across the 200 MSD time integration steps. This gradient pathway allows physics errors to directly influence parameter predictions, enabling the network to learn biomechanical properties without any supervision on the mechanical parameters, the interaction force, or the boundary conditions. The physics loss transforms the problem from supervised parameter regression, which would require expensive ground-truth measurements, to unsupervised deformation prediction, which requires only observable video data, and it does so in an amortized form: the cost of simulation is incurred during training, so that at inference, the parameters follow from a single forward pass of the network rather than from an optimization repeated for every frame, as would be required were the simulator treated as a black box.

The parameter regularization loss enforces soft constraints on parameter ranges, penalizing predictions that approach or violate physically unreasonable bounds:

(16) Lparam_regP=EDt ∑p ∈ks, kd,Fmag(max 0, pmin−p2+max0, p− pmax2)

Here, *p* denotes any of the three predicted parameters, and $\;p^{min}\;$and $p^{max}$ are its admissible bounds, set to [0, 1] for $k_{s}$ and $k_{d}$ and [−10, 0] for $F_{mag}$. The penalty is one-sided in each direction: it vanishes identically for any prediction falling inside the admissible interval and grows quadratically outside it, so that it does not bias the estimate within the admissible range. Its role is complementary to the bounded activations of the parameter estimation head described in Section 3.4.1: the activations enforce these ranges architecturally, whereas $L_{param\_reg}\;$states them as an explicit optimization-level constraint that does not depend on the choice of output nonlinearity and would remain effective were that parameterization changed. The comparatively low weight assigned to this term reflects its auxiliary function: it acts as a boundary safeguard rather than as an objective competing with the physics consistency and reconstruction terms. The loss weights reflect the physical role of each term: $\lambda_{cyc}$ = 10.0 enforces temporal coherence as the primary structural constraint; $\lambda_{GAN}$ = $\lambda_{ID}$ = 1.0 operate at the baseline scale for perceptual realism and identity regularization, respectively; $\lambda_{phys}$ = 2.0 prioritizes physics consistency over pure adversarial realism (the physics loss operates on displacement quantities numerically smaller than pixel-level residuals; so, moderate upweighting maintains effective gradient flow through the MSD simulator). The specific values were validated empirically on the held-out validation set.

#### 3.4.3. Training Procedure

Each training sample consists of a consecutive depth map pair $\left(D_{t};D_{t+1}\right)$, the triangulated mesh $M_{t}=\;\left(V_{t,\;},\;F_{t}\right)$ extracted from Dt following the geometric pipeline (Section 3.2.2), and the contact information (${idx\;}^{*},{n\;}^{*},{g\;}^{*})$ computed through nearest-neighbor search between the grasper and gallbladder point clouds. The training workflow proceeds through two parallel pathways.

In the reconstruction pathway, the current depth map $D_{t}$ is passed through the encoder E to extract feature representations. The parameter estimation network *p* then processes these features to predict the physical parameters $\theta ={\left[k_{s},\;k_{d},\;F_{mag}\right]}^{T}.$ The predicted parameters θ are injected into the encoded features and fed into the generator G to produce the predicted next-frame depth map, $\;D_{t+1}$, establishing the link between parameter conditioning and depth map prediction.

In the physics pathway, the predicted parameters, θ, are fed into the differentiable MSD simulator $\Phi_{MSD}\;$ along with the mesh geometry, $M_{t}$, and contact information to compute the expected displacement, $d_{sim}$. The observed displacement, $d_{obs}$, is extracted by comparing the contact point positions between consecutive depth maps, $D_{t}$ and $D_{t+1}$. Critically, gradients flow through two distinct pathways back to the parameter estimation network: (1) through the conditional generator from reconstruction errors, and (2) through the differentiable MSD simulator from physics consistency errors. The latter pathway involves backpropagation through time across 200 MSD integration steps, enabling the network to learn how parameter variations affect tissue deformation dynamics. This dual-pathway gradient flow ensures that predicted parameters must simultaneously produce realistic depth map transformations and physically consistent deformations.

**Figure 2:**
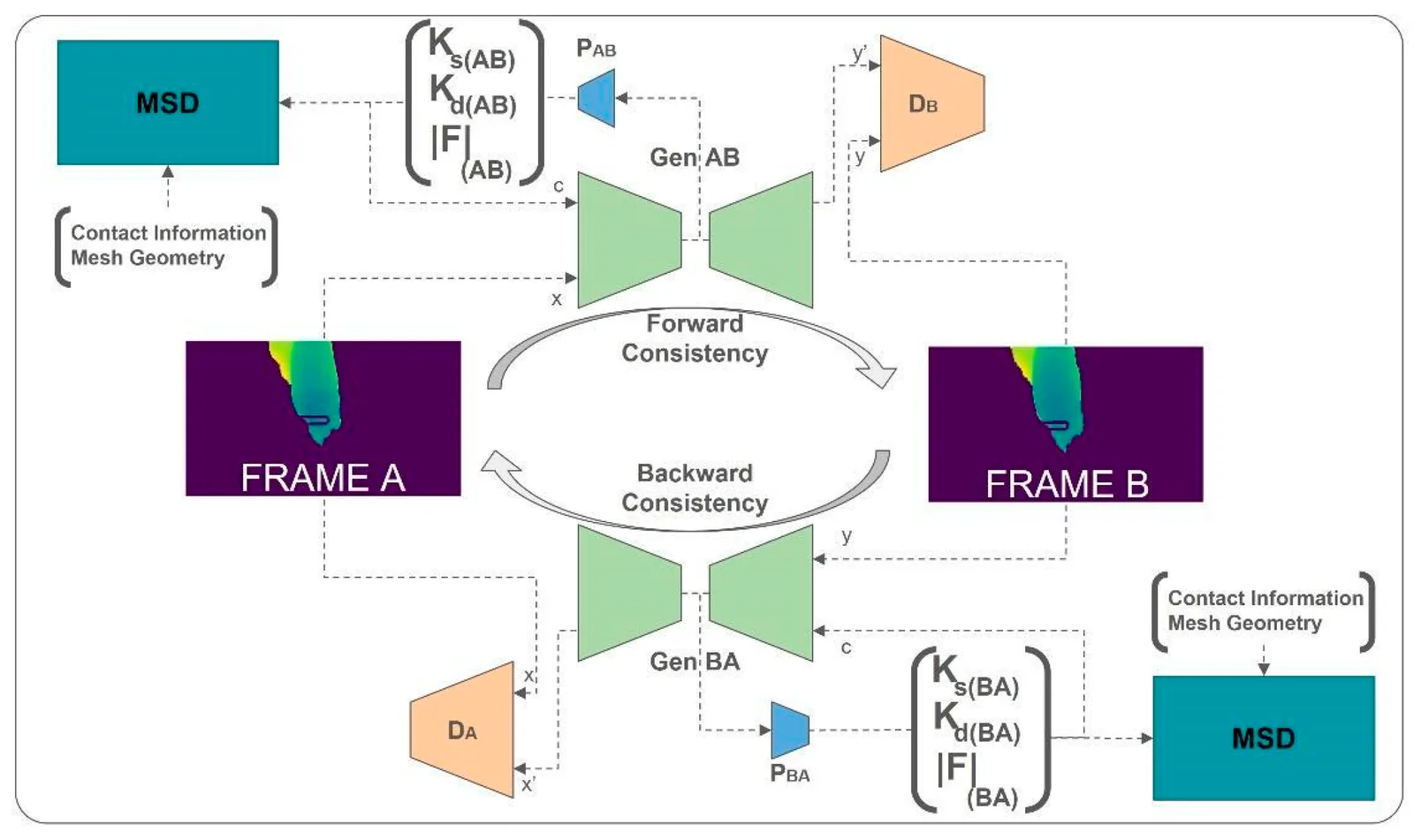
Training framework overview. Two generators (GAB, GBA) operate on consecutive depth maps (Frame A = $D_{t}$, Frame B = $D_{t+1}$). Each generator has a dual role: the parameter head ($P_{AB}$/$P_{BA}$) estimates biomechanical parameters ($k_{s},\;k_{d},\;|F|$), which condition the generation of the predicted next-frame depth map. Discriminators ($D_{A}$, $D_{B}$,) enforce adversarial realism, while forward–backward consistency loops ensure cycle-consistent transformations. The estimated parameters, together with contact information and mesh geometry, are fed to differentiable MSD simulators that provide physics-based supervision by comparing simulated and observed tissue displacement. Gradients flow back through both pathways, enabling unsupervised learning of tissue mechanical properties.

The discriminator update follows standard adversarial training: it is trained to distinguish real depth maps $D_{t+1}$ from generated depth maps $\hat{D_{t+1}}$, with gradients prevented from flowing back to the generator through detachment operations. The complete training configuration is summarized in Figure 2.

#### 3.4.4. Inference Configuration

At inference time, the trained network operates in a streamlined mode. The inference pipeline processes each incoming video frame sequentially: first, depth estimation and preprocessing generate the depth map, $D_{t}$, mesh, $M_{t}$, and contact information, (${idx\;}^{*},{n\;}^{*},{g\;}^{*})$; second, the parameter estimation network extracts the physical parameters, $\;\theta ={\left[k_{s},\;k_{d},\;F_{mag}\right]}^{T}$, through a single forward pass; third, the MSD simulator computes the expected deformation $d_{sim}\;$using the extracted parameters. Only the parameter estimation pathway (encoder and parameter head) is executed during inference. The conditional generation pathway used during training is not required for deployment, reducing computational overhead. The MSD simulation runs in forward-only mode without maintaining a computational graph.

#### 3.4.5. Parameter Identifiability Considerations

The three parameters ($k_{s},\;k_{d},\;F_{mag}$) are estimated jointly from depth observations without direct supervision, which raises the question of whether the observation model determines them separately. The structure of Equation (9) makes explicit what is resolved and what is not. Under static loading, where the acceleration and velocity vanish, the dynamic equilibrium reduces to $x_{eq}=\;F_{mag}\;/k_{s}$: only the ratio of force to stiffness is determined, the two quantities are individually undetermined, and the damping coefficient does not enter the observation at all. This degeneracy resolves once the transient response is observed. The homogeneous dynamics of Equation (9) are characterized by the natural frequency $\omega_{n}$ = $\sqrt{\left(k_{s}/m\right)}$ and the damping ratio $\zeta =k_{d}/2\sqrt{\left(k_{s}/m\right)}$), so that a transient trajectory determines ks from the oscillation frequency and kd from the rate of decay, with $F_{mag}\;$ then following from the equilibrium offset. Because the nodal mass m is fixed a priori (Section 3.3) rather than estimated, this parameterization is not subject to the scale ambiguity that would arise if mass, stiffness and force were inferred jointly. Identifiability in our setting therefore rests on a specific and verifiable condition, namely that the observed sequences contain transient dynamics rather than quasi-static contact alone. Two structural mechanisms in the framework ensure that this condition is met and further constrain the feasible parameter space.

First, the predicted parameters are anchored to observed tissue deformation through two independent supervision signals. The physics loss (Equation (14)) directly measures the discrepancy between MSD-simulated deformation and the observed contact displacement dobs, which is a physical recording of what the tissue did under real surgical forces. The conditional generation pathway enforces a second, independent observational constraint: the predicted parameters condition the generation of the next depth map, evaluated against the observed frame through both cycle consistency and adversarial losses. A degenerate parameterization that minimizes the physics loss by trading off $k_{s}$ against $F_{mag}\;$ would produce an incorrect depth prediction, increasing the reconstruction loss. This cross-coupling between physics fidelity and observational reconstruction creates a closed feedback loop in which only a parameter configuration consistent with the true biomechanical state satisfies both supervision signals simultaneously.

Second, the training sequences capture both active contact phases (tool pressing on tissue) and passive release phases (tool disengaged). During the passive release, $F_{mag}\;$ drops to zero and the tissue dynamics are governed solely by $k_{s},\;k_{d}\;$ through the homogeneous MSD equation. This temporal structure provides a natural decoupling signal that constrains the mechanical parameters independently of the applied force, reducing collinearity among the three quantities. This passive phase is precisely the regime identified above: with $F_{mag}$ = 0, the observed decay is governed by $\omega_{n}$ and ζ alone, so that ks and kd are determined by the shape of the free response independently of the force estimate. Section 4.2 documents this behavior in the test sequences. Future work should include controlled synthetic experiments with known ground-truth parameters to verify whether the estimated quantities correspond to unique physical solutions.

### 3.5. Implementation Details

The framework was implemented in PyTorch (v. 2.10). The MSD simulator is written as a differentiable module, so that gradients of the physics loss propagate to the parameter estimation head through automatic differentiation; gradient checkpointing was applied across the integration horizon to bound memory consumption during backpropagation through the 200 time steps. The two generators share a single Adam optimizer with learning rate 2 × 10^−4^ and momentum coefficients β = (0.5, 0.999), while each discriminator is optimized separately with the same settings. Training was run for 100 epochs with a batch size of 2, the latter necessitated by the memory footprint of the unrolled simulation, and the reported model is the checkpoint achieving the lowest validation physics-consistency loss. Experiments were tracked with MLflow, with checkpoints stored every 10 epochs. All experiments were conducted on a single NVIDIA GeForce RTX 3060 GPU with 12 GB of VRAM, and the inference latency reported in Section 4.3 was measured on the same hardware.

## 4. Results and Discussion

The proposed physics-informed generative framework was evaluated using a subset of 1190 frames from the CholecSeg8k dataset, focusing on tool–tissue interaction sequences during laparoscopic cholecystectomy. The evaluation focused on three main pillars: parameter estimation accuracy assessed through indirect simulation consistency, physical consistency of the dynamic tissue response, and computational sustainability for intraoperative use.

It is worth noting that quantitative comparison with supervised force estimation methods is methodologically precluded by structural differences in sensing modality, supervision regime, and evaluation substrate. Vision-based force estimation methods across the literature [13,14,15,16] train networks to regress force from visual input using synchronized measurements from load cells, force-sensing trocars, or FBG sensors, and are consequently evaluated on benchtop platforms, phantoms, ex vivo tissue, or instrumented robotic platforms. This structural incomparability is recognized at the field level. The systematic review by Han and Dou [22] explicitly cautions that reported accuracy values across intraoperative deformation modeling methods should be considered as references for algorithm performance rather than sole criteria for assessment, given the influence of deformation extent, organ type, observation modality, and experimental substrate. Wang et al. [16] similarly note that prior temporal-network-based methods could not be fairly compared with their structured-light approach because of analogous modality differences. The proposed framework therefore occupies a distinct niche: unsupervised biomechanical parameter recovery from clinical-grade intraoperative video, a capability that sensor-supervised approaches cannot operate in by design.

### 4.1. Quantitative Validation and Statistical Analysis

We note that absolute validation of the estimated parameters against ground-truth force measurements is not feasible on this dataset, as no intraoperative force sensors were used during data collection. The evaluation is therefore framed as indirect validation through simulation consistency, which is the appropriate metric given the unsupervised nature of the framework. This is consistent with the evaluation strategy adopted across the broader field of intraoperative deformation modeling, where the systematic review by Han and Dou [22] explicitly notes that reported accuracy values are subject to the extent of deformation, specific organ, intraoperative observation modality, and experimental substrate (in silico, phantom, ex vivo, in vivo).

We compared the simulation error obtained with PINN-predicted parameters against a fixed-parameter reference configuration, in which the internal stiffness and damping are held at the literature values $k_{s}\;$ = 5.34 and $k_{d}$ = 0.127 [19] rather than estimated from the scene. All other elements of the simulation are identical between the two regimes, whose constants remain fixed throughout. These literature values were characterized for bulk tissue and are applied here to the edge-wise internal stiffness of the mesh; so, the comparison is between an adaptive and a fixed parameterization of the same simulator rather than a validation of the estimated values against a physically equivalent reference. As summarized in Table 2, the PINN-based adaptation outperforms static literature values across all 176 evaluation frames. MSE 95% confidence intervals are also reported.

A Wilcoxon signed-rank test confirmed that the improvement achieved by the PINN is statistically robust on the held-out sequence (W = 3658, *p* < 10^−6^), a result corroborated by the paired *t*-test (t = 5.16, *p* = 1.1 × 10^−6^). Bias-corrected and accelerated bootstrap confidence intervals, computed over 10,000 resamples, are reported in Table 2: the mean simulation error is 0.00467 (95% CI [0.00286, 0.00684]) under the fixed-parameter configuration and 0.00428 (95% CI [0.00256, 0.00638]) under PINN estimation. The calculated Cohen’s d of 0.390 indicates a consistent, small-to-moderate effect.

While the absolute MSE reduction is modest, two considerations support the use of the learning framework over static parameters. First, fixed literature values are population-level estimates that do not account for inter-patient variability in tissue mechanical properties; the PINN adapts to the specific tissue observed intraoperatively. Second, as shown in Section 4.2, the learned parameters recover physically correct viscoelastic settling behavior that fixed parameters fail to reproduce, which is the more clinically relevant criterion for haptic feedback and safety monitoring: a system using fixed literature parameters may achieve similar average MSE while producing qualitatively incorrect dynamics (e.g., underdamped oscillations), which would be clinically problematic.

### 4.2. Analysis of Dynamic Tissue Response

**Figure 3:**
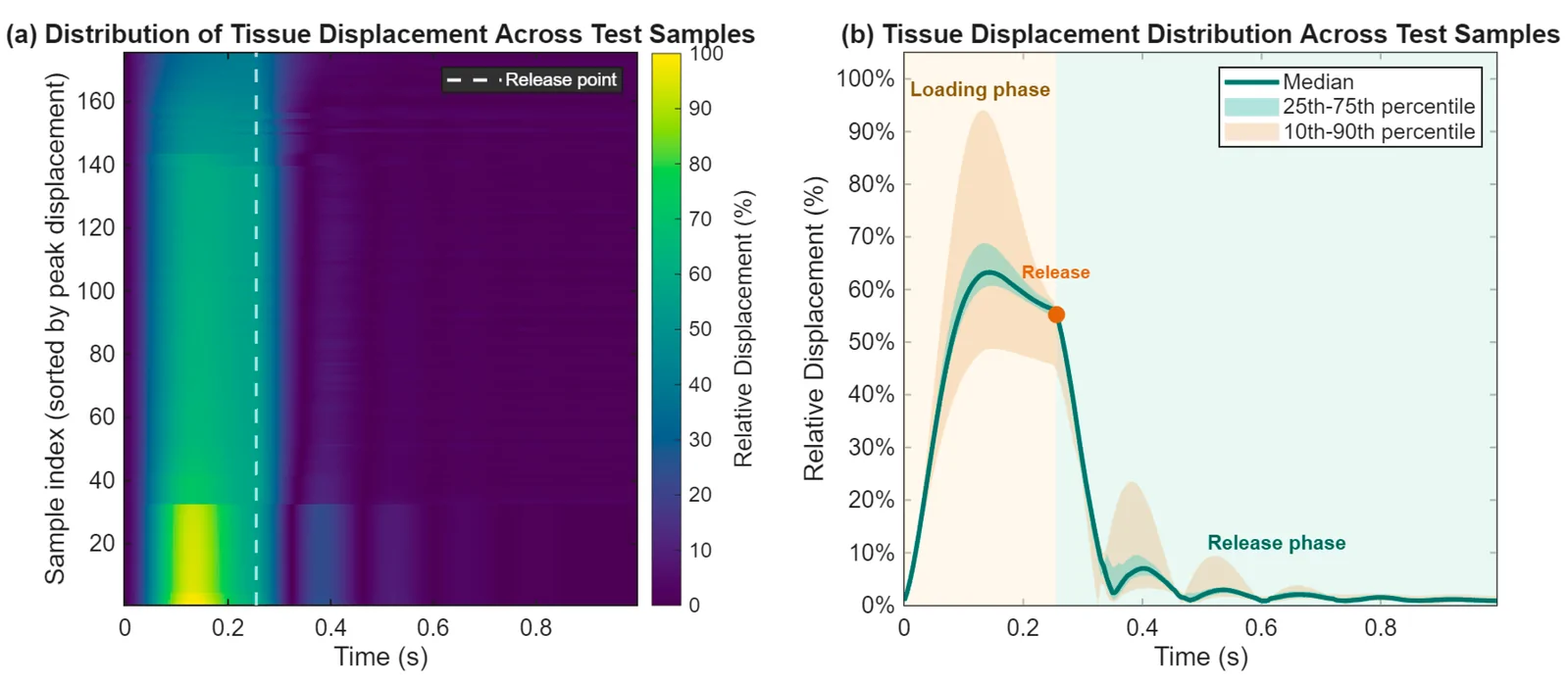
Oscillation analysis and viscoelastic behavior. (**a**) Heatmap displaying the distribution of relative tissue displacement across all test samples, sorted by peak displacement, with the dashed line indicating the release point. (**b**) Fan chart of tissue displacement distribution over time showing the median trace (dark green line), the 25–75th percentile range (light green shaded area), and the 10–90th percentile range (tan shaded area). The plots distinguish between the loading phase and the release phase, capturing post-contact damped oscillations that reflect the specific viscoelastic properties estimated by the model.

Figure 3 illustrates the temporal displacement of the gallbladder tissue during deformation and subsequent release. The mean trace shows a relative displacement increase, reaching approximately 65% of the global peak during manipulation. The Release Point (at approximately 0.25 s) marks the transition from active tool-driven deformation to passive dynamic response.

Figure 3 illustrates the temporal displacement of the gallbladder tissue during deformation and subsequent release across the test samples. As shown in the fan chart (Figure 3b), during the loading phase, the median trace shows a relative displacement increase, reaching approximately 63% of the global peak during manipulation, while the overall distribution boundaries are captured by the 25–75th and 10–90th percentiles. The Release Point (at approximately 0.25 s) marks the sharp transition from active tool-driven deformation to passive dynamic response. This transition is equally visible across all individual sequences in the heatmap (Figure 3a), which sorts the samples by their peak displacement to highlight global behavioral consistency.

Following the release, the tissue exhibits viscoelastic behavior characterized by distinct damped oscillations within the release phase. The model returns to equilibrium (displacement < 5%) within 0.9 s, demonstrating that the PINN correctly learned the damping coefficient $k_{d}$ necessary to dissipate kinetic energy in a physically plausible manner. This post-release behavior also confirms that the test sequences contain the free-vibration regime on which parameter separability depends (Section 3.4.5): with the tool disengaged, the observed decay is governed by the natural frequency and damping ratio alone, which determine $k_{s}$ and $k_{d}\;$ independently of the estimated force. The tight percentile bands during the release phase suggest that the framework provides stable and robust mechanical predictions despite the inherent geometric and structural variability in surgical scenes.

Across all test frames, the network estimated mean values of $k_{s}=\;$0.508 ± 0.0027, $k_{d}$ = 0.09 ± 0.0162 and $\;F_{mag}$ = 7.79 ± 0.1841, expressed in the normalized units introduced in Section 3.3. The dispersions are narrow, indicating that the estimator converges to a consistent mechanical characterization across the sequence. Since these quantities are defined up to a global-scale factor, they are not directly comparable with absolute values obtained by ex vivo characterization; the evidence that they are physically meaningful comes instead from the dynamic behavior they produce, namely the damped return to equilibrium documented above, which fixed parameters fail to reproduce.

### 4.3. Computational Performance and Deployment Considerations

Deployment viability in intraoperative contexts requires inference latency compatible with real-time surgical navigation and robotic control loops. Parameter estimation from a single RGB/depth input required on average 1.67 ms per frame, with the fastest recorded passes being completed in 0.83 ms. The corresponding throughput ceiling of approximately 600 Hz exceeds standard endoscopic video frame rates (30–60 fps) by more than an order of magnitude. The cost of the complete pipeline, however, is dominated by the depth estimation front end: measured on the same hardware, DepthPro required approximately 921 ms per frame in half precision, against 1.67 ms for parameter estimation, so that end-to-end inference proceeds at roughly one frame per second. Moreover, this cost is independent of the input resolution, as the model operates internally at a fixed 1536 × 1536 resolution [6]. The characterization proposed here therefore adds 1.67 ms to a pipeline whose rate is set entirely by its depth estimator: for any system already producing monocular depth, mechanical parameter estimation is obtained at negligible marginal cost, and any improvement in monocular depth estimation translates directly into the end-to-end rate. The differentiable simulator does not lie on this path: gradients propagate through it while the network is trained, whereas at deployment, the parameters follow from a single forward pass. Where forward prediction of the deformation is required, a rollout of 1.0 s of simulated dynamics over 200 steps, on test meshes of approximately 14,800 vertices, required 135.9 ms, advancing some seven times faster than real time. For integration into a robotic platform, what matters is the rate each consumer of the estimate requires. The quantity produced here is a mechanical characterization of tissue, and such characterization varies slowly: stiffness varied by well under one percent across the test sequence. Visual overlays and haptic loops are therefore parameterized by the estimate rather than gated on its arrival. All measurements were obtained on a consumer-grade GPU with 12 GB of memory, with the depth model being reported by its authors at approximately 0.3 s per frame on datacenter-class hardware [6], which would reduce the dominant cost substantially.

## 5. Limitations and Future Work

Several limitations of the current implementation warrant explicit acknowledgment. The current pipeline relies on semantic segmentation to isolate tool and tissue depth representations. In the present implementation, this uses the full 13-class annotations provided by CholecSeg8k, which would not be available in unlabeled intraoperative video. However, the architecture technically requires only a binary tool-versus-tissue separation, which could be achieved by a lightweight binary segmentation or depth-discontinuity-based approach. The landscape of real-time segmentation foundation models has evolved rapidly, with mobile-optimized variants such as MobileSAM [36] demonstrating real-time inference speeds compatible with surgical pipelines, and domain-adapted models such as MCP-MedSAM [37] providing further reduction in the gap to clinical deployment. Future work will investigate these as drop-in replacements to reduce deployment complexity, and quantify how segmentation error propagates into the estimated parameters.

Depth estimation accuracy represents a further source of uncertainty. Depth maps produced by DepthPro may introduce scale errors in regions of low texture, specular reflection, or smoke artifact, which propagate to the contact geometry reconstruction and affect the reliability of the observed displacement signal fed to the physics loss.

Contact detection reliability is additionally constrained by instrument occlusion. Partial occlusion of the contact region due to instrument overlap, blood, or irrigation fluid may prevent reliable contact point detection, introducing noise into the geometric pipeline inputs.

The absence of intraoperative force measurements precludes absolute validation of the estimated parameters; evaluation therefore follows an indirect simulation-consistency protocol.

Formal identifiability of the three parameters ($k_{s},\;k_{d},\;F_{mag}$) from depth observations alone has not been established. As discussed in Section 3.4.5, the framework incorporates two complementary mechanisms that constrain the parameter space, but a rigorous identifiability analysis requires controlled synthetic experiments with known ground-truth parameters. Future work should generate synthetic data from the MSD simulator with known $k_{s},\;k_{d}\;and\;F_{mag}$ values and verify whether the framework recovers these quantities with quantifiable accuracy.

Furthermore, the MSD formulation assumes linear viscoelastic behavior and spatially uniform parameters. The framework assumes spatial homogeneity of mechanical parameters within the contact region, which may not hold in pathological tissue or at anatomical boundaries. While soft-tissue mechanics are more accurately described by nonlinear hyperelastic constitutive models, the MSD approximation was retained for its computational efficiency and real-time compatibility, which are essential requirements for intraoperative deployment.

While direct quantitative comparison with supervised force estimation methods is precluded by the structural differences described in Section 4, future work could investigate hybrid approaches in which sparse force supervision signals, available in structured laboratory settings, are used to calibrate or validate the unsupervised parameter estimates, potentially bridging the two paradigms.

The current evaluation is limited to cholecystectomy on a single dataset. Generalization to other anatomical structures and surgical procedures represents an important future direction, particularly given the known variability in tissue mechanical properties across organ types and pathological states.

## 6. Conclusions

This paper presented a physics-informed generative framework for unsupervised biomechanical parameter and interaction force estimation from laparoscopic depth maps. By embedding a differentiable MSD simulator into a neural generative architecture, the framework estimates scene-adapted stiffness ($k_{s}$) and damping ($k_{d}$), and tool–tissue interaction force ($F_{mag}$) directly from surgical video, without requiring any force-sensor, displacement, or boundary-condition supervision.

The complete pipeline, from monocular metric depth estimation via DepthPro, through a 3D geometric contact detection pipeline, to PINN-based parameter estimation with a five-term physics-adversarial loss, estimates the mechanical parameters at 1.6 ms/frame, a negligible addition to the monocular depth front end, which sets the end-to-end rate of the pipeline. The experimental results on CholecSeg8k confirm that PINN-estimated parameters significantly outperform static baseline values (*p* = 2.49 × 10^−8^, Cohen’s d = 0.374), and dynamic tissue response analysis confirms accurate capture of viscoelastic settling behavior with equilibrium recovery within 0.9 s of tool release.

The estimated parameters directly enable stiffness-aware haptic rendering [38], intraoperative safety monitoring [2,13,25], and patient-specific surgical simulation initialization [39]. In haptic rendering, the inferred $k_{s}\;$ can modulate force feedback proportionally to local tissue compliance at latencies compatible with current surgical interfaces (500 Hz–1 kHz) [38], without dedicated force transducers. The estimated $F_{mag}$ provides a continuous sensor-free proxy for tool–tissue interaction forces monitorable against established safety thresholds [2], offering a clinically deployable alert mechanism for excessive force application [13,25]. Beyond safety monitoring, continuous force and stiffness estimates could inform intraoperative decision support systems and provide objective metrics for surgical skill assessment, complementing existing kinematic and video-based approaches. Patient-specific simulation initialization with intraoperatively estimated parameters addresses a recognized limitation of current simulators, where fixed population-level tissue values reduce ecological validity relative to the individual patient [39]. Collectively, these applications address the core challenge of operating without tactile feedback in MIS. The framework represents a step toward physically grounded, sensor-free tissue characterization from standard laparoscopic video, with identifiability analysis and broader anatomical validation as the primary directions for future work. Future work will explore nonlinear constitutive tissue models and finite-element-based differentiable simulation to improve physical fidelity, uncertainty-aware parameter estimation to quantify prediction reliability, and multimodal sensing integration to strengthen observational grounding.

## Figures and Tables

**Table 1:** Physical simulation parameters used in the MSD simulator.

Symbol	Value	Unit	Definition
**m**	0.001	kg	Point mass assigned to each mesh vertex
**Δt**	0.005	s	Numerical integration timestep
**T**	200 × Δt	s	Total simulation duration over 200 integration steps
**Integrator**	Explicit (semi-implicit) Euler: $v\;+=(\frac{F}{m})\cdot dt$, x +=v·dt	—	Semi-implicit Euler scheme: velocity updated with current forces, position updated with new velocity
**ST**	1	s	Simulation time

**Table 2:** Comparative analysis of simulation error between the fixed-parameter reference configuration and PINN estimation, on the held-out test sequence (n = 176 consecutive frame pairs). Errors are expressed in the normalized units of Section 3.3. Confidence intervals are bias-corrected and accelerated bootstrap intervals over 10,000 resamples.

Metric	Default Params	PINN Params
**MSE (mean ± SD)**	0.00467 ± 0.01361	0.00428 ± 0.01308
**MSE, 95% CI**	[0.00286, 0.00684]	[0.00256, 0.00638]

## Data Availability

The CholecSeg8k dataset used in this study is publicly available at https://arxiv.org/abs/2012.12453 (accessed on 24 October 2025).

## Abbreviations

The following abbreviations are used in this manuscript:

AR	Augmented reality
AI	Artificial intelligence
FEM	Finite element method
fps	Frames per second
GAN	Generative adversarial network
KD-tree	K-dimensional tree (spatial data structure)
LSGAN	Least-squares generative adversarial network
MIS	Minimally invasive surgery
MSD	Mass–spring–damper
PINN	Physics-informed neural network
SD	Standard deviation
ViT	Vision transformer
ks	Spring stiffness coefficient
kd	Damping coefficient
F_mag_	Interaction force magnitude
